# Newborn and infant hearing screening at primary healthcare clinics in South Africa designated as National Health Insurance pilot sites: An exploratory study

**DOI:** 10.4102/sajcd.v69i1.840

**Published:** 2022-01-26

**Authors:** Amisha Kanji

**Affiliations:** 1 Department of Speech Pathology and Audiology, Faculty of Humanities, University of the Witwatersrand, Johannesburg, South Africa

**Keywords:** newborn hearing screening, integration, primary healthcare, National Health Insurance, South Africa

## Abstract

**Background:**

Primary healthcare (PHC) is the first point of entry, providing basic services to individuals. South Africa is in the process of re-engineering its PHC as part of National Health Insurance (NHI) plans to ensure universal healthcare coverage.

**Aim:**

This study aimed to establish whether newborn and infant hearing screening (NIHS) could be integrated into the re-engineering process of the PHC as part of the NHI framework.

**Setting:**

The NHI pilot clinics in five provinces in South Africa.

**Methods:**

A non-experimental, descriptive, cross-sectional survey research design was adopted. Questionnaires were sent to nursing managers, unit managers or acting managers at PHC facilities. Nineteen of these self-administered questionnaires were completed. Data were analysed using descriptive statistics.

**Results:**

Immunisation services were the most common type of service offered at the clinics. Over a quarter of the respondents indicated that NIHS services were offered at their facility in the form of universal NIHS. Equipment was limited with a lack of valid and reliable screening measures. Only 2 (11%) respondents indicated budgetary resources. Follow-up and referral pathways were reported by 10 (53%) respondents, which did not include an audiologist.

**Conclusions:**

There is a need for careful and systematic planning in terms of early hearing detection programmes at PHC level. Planning needs to commence with considerations of who will perform NIHS, training of these personnel by audiologists and the role of the audiologist within the teams outlined in the NHI Bill.

## Introduction

Healthcare in South Africa comprises both public and private sectors. This two-tiered system is considered intricate, with an additional, hierarchical structure within the public healthcare sector. This hierarchical structure in the public sector requires that advanced levels of care may only be accessible following assessment and referral from the respective lower levels, and is based on the implemented primary healthcare (PHC) approach (Cullinan, 2006; Mahlathi & Dlamini, 2015).

Primary healthcare is the first level of care and entry for individuals into the healthcare system, which is accessible at PHC clinics and community health centres (CHCs). The services at these healthcare facilities include maternal and child care, immunisations, family planning and care for chronic illnesses, usually managed by nurses with advisory support and regular visits by medical practitioners and other relevant healthcare specialists (Bresick, Von Pressentin, & Mash, 2019; Cullinan, 2006). Such services can more broadly be categorised as preventative, promotional, curative and rehabilitative in nature (Cullinan, 2006; Schellack, Meyer, Gous, & Winters, 2011).

An earlier review of the quality of services was conducted at 16 clinics in Johannesburg. Poor quality child health services for children who are ill was observed in relation to waiting periods, staff skills, triage and promotional, prevention and curative care. This was further accompanied by insufficient developmental assessment (Thandrayen & Saloojee, 2010). Of the 16 clinics, inconsistency was noticed in terms of immunisation and rehabilitation in 14 and 5 clinics, respectively. The Integrated Management of Childhood Illnesses (IMCI) approach was only utilised in 3 clinics, despite 12 having had an IMCI-trained professional. These findings by Thandrayen and Saloojee (2010), although not the most recent, serve as important considerations when deciding on an appropriate and feasible model for early detection of hearing loss, particularly in PHC contexts.

As PHC sites provide an extensive package of basic services, which includes maternal and child health, newborn and infant hearing screening (NIHS) can be argued to fall within this service. Early identification of hearing loss involves NIHS using various objective screening measures such as otoacoustic emissions or automated auditory brainstem response and is a beneficial preventive strategy that forms part of early hearing detection and intervention (EHDI). The EHDI guidelines of the Health Professions Council of South Africa (HPCSA) (2018) highlight six principles as the foundation to effective EHDI systems in South Africa. These principles include (1) access to hearing screening using physiological measures, including PHC clinics and Midwife Obstetric Units (MOUs) as possible contexts for access to these services in order to ensure screening by 1 month of age, (2) access to an effective referral system to ensure prompt diagnosis of hearing loss by 3 months or 4 months if NIHS is aligned to immunisation visits at PHC facilities and (3) access to intervention services following diagnosis of hearing loss before 6 months or 8 months of age for infants who were enrolled in NIHS programmes aligned with immunisation visits at PHC facilities (HPCSA, 2018).

The PHC clinics have been suggested as a setting for NIHS by the HPCSA with the justification that this level of care allows a larger reach, resulting in increased coverage of screened infants and a higher attendance at follow-up appointments (HPCSA, 2007, 2018; Swanepoel, Hugo, & Louw, 2006). Khoza-Shangase and Harbinson (2015) also proposed the incorporation of the MOU 3-day assessment clinic as a NIHS platform in order to ensure improved coverage rates. Although an earlier pilot study conducted at two immunisation clinics indicated the potential implementation of hearing screening at these sites with a good coverage rate and collaborative nursing personnel in most instances, there were a number of documented barriers to successful implementation (Swanepoel et al., 2006).

More recently (in 2016), community-based NIHS programmes have been initiated within the PHC platform of the Western Cape. Hearing screening was administered by trained nurses using a two-stage distortion product otoacoustic emission protocol at Maternal and Child Health Care clinics over a period of 19 months (De Kock, Swanepoel, & Hall, 2016). The return rate for follow-up appointments was generally good but differed between clinics. However, coverage rates (32.4%) were poor across clinics and did not meet the criteria specified by the key benchmarks in the HPCSA (2007) EHDI position statement (Friderichs, Swanepoel, & Hall, 2012). Although not specifically investigated, the authors acknowledge that findings may have been affected by the high staff turnover at some clinics and the use of nursing staff as screeners who experienced difficulty including screening as part of their other regular priority tasks (Friderichs et al., 2012). Although this study utilised trained nursing staff, it lacked information about the existing resources and whether or not the setting is ready for the provision of these services. Such information is necessary in order to facilitate planning for sustainable service provision. Another study conducted at MOUs reported good follow-up return rates (De Kock et al., 2016). These studies have been based on measuring the outcomes in relation to specific key indicators such as coverage rates, follow-up return rates and referral rates. However, there has been limited research into whether PHC clinics are actually equipped and ready to take on NIHS considering the proposed National Health Insurance (NHI) framework with a particular focus on dealing with infants at risk for hearing loss – who may require more prompt services.

Other studies related to the provision of NIHS have been conducted at immunisation clinics in North West and Gauteng (Petrocchi-Bartal & Khoza-Shangase, 2014a, 2016). Information gathered through interviews with nursing staff highlighted that clinic managers were eager to have hearing screening incorporated into their workload provided that specific barriers related to aspects such as budget and equipment resources were addressed (Petrocchi-Bartal & Khoza-Shangase, 2014a, 2016). The availability of current resources needs to be carefully considered within the context of each province before implementing NIHS programmes nationally, particularly in view of PHC re-engineering within the proposed NHI framework. A study exploring the perception of professional nurses in the private sector with regard to NHI was mixed. Whilst nurses acknowledged the purpose of the NHI, there were concerns regarding the current shortage of personnel, inadequate resources and a lack of stakeholder engagement and transparency with regard to implementation (Molokomme, Seekoe, & Goon, 2018). Similarly, general practitioners at one of the Tshwane district pilot sites expressed frustration at the lack of infrastructure and equipment (Surender, Van Niekerk, & Alfers, 2016). The lack of communication and exclusion in NHI policy formulation was also expressed in recent findings from in-depth interviews with public service managers in the Johannesburg health district (Murphy & Moosa, 2021).

The re-engineering of PHC services will centre around community-based outreach and home-based service provision aimed at promotion, prevention and quality curative and rehabilitative services (DoH, 2011). According to the recent NHI Bill, these services will be provided through various means, namely district clinical specialist teams, integrated school health services, municipal ward-based PHC outreach teams and contracting of private healthcare professionals at a non-specialist level (DoH, 2015, 2019). The municipal ward-based PHC outreach teams will provide services within specified areas, led by a nurse associated with a PHC facility (DoH, 2011, 2015; Naidoo, 2012). These teams will foster community engagement in terms of the detection of health problems associated with the risk for disease and need of preventative, curative and rehabilitative services. Health promotion education is also another role that will be played by these teams (DoH, 2011, 2015; Naidoo, 2012). Based on the assessments during home visits, community healthcare workers would need to make appropriate referrals to a PHC facility. Hence, the role of the PHC nurse is important to the early identification of hearing loss in newborns or infants.

Piloting of the NHI suggests the need to explore NIHS programmes within this proposed healthcare funding model. Prior to the actual implementation of such programmes, it is important to gather the necessary information to determine the preparedness and readiness of this level of healthcare for provision of NIHS services. According to Myezwa and Van Niekerk (2013) each medical profession, including rehabilitation professionals need to assess their service delivery in terms of quality, financial allocation of resources and personnel, and their response to the burden of disease. Gathering information related to the availability of services and resources at the defined NHI PHC pilot clinics will assist in the planning and clinical implementation phases of NIHS programmes at PHC level within the proposed NHI framework. This is particularly important considering that insufficient planning, resource shortages, inconsistent communication and inadequate coordination were factors that hindered the success of NHI phase 1 implementation within pilot districts (Genesis, PwC, CHP, & Insight, 2019).

This study aimed to describe the current resources available for NIHS for future NHI implementation by (1) describing the services offered for early detection of hearing loss in newborns and infants, (2) determining the resources available for NIHS, (3) describing the protocols and procedures for screening of well babies and high-risk newborns and infants and (4) describing the follow-up and referral pathways.

## Methods

### Study design

A non-experimental, descriptive, cross-sectional survey research design was adopted.

### Setting

The study comprised seven NHI pilot PHC clinics in five provinces in South Africa from which permission was obtained. Within these five provinces, seven PHC clinics have been listed as designated NHI sites in the North West Province, five are designated NHI sites in the Northern Cape, six in Mpumalanga, six in Limpopo and eight in the Eastern Cape.

### Sampling and sample

The study used a non-probability, purposive sampling strategy. Participants comprised nursing staff who are unit managers, clinic managers and acting managers at various PHC facilities in South Africa, particularly those facilities within the NHI pilot districts.

### Data collection

A questionnaire was sent to nursing managers, unit managers or acting managers at various PHC facilities in South Africa, particularly those PHC facilities that fall within the pilot districts for NHI. The questionnaire comprised 23 closed-ended and 7 open-ended questions related to services offered for early detection of hearing loss in newborns and infants, resources available for newborn hearing screening, protocols and procedures for screening of well babies and high-risk newborns and infants, as well as follow-up and referral pathways. The questionnaire was adapted from other similar studies (Khoza-Shangase, Kanji, & Ismail, 2021; Petrocchi-Bartal & Khoza-Shangase, 2016).

Questionnaires were either distributed electronically or via courier, depending on the preferred method of dissemination communicated by each of the facilities. Approximately 25 questionnaires were sent via courier services and 6 were sent electronically. Reminders were sent in an effort to improve the response rate.

### Data analysis

Data were analysed using descriptive statistics.

### Ethical considerations

Ethical clearance (reference number: M170859) was obtained from the University’s Medical Ethics Committee. Permission to conduct the study in various provinces was obtained through application on the National Health Research Database (NHRD). Of the nine provinces, five provided permission via the NHRD. Of the nine provinces, permission was obtained from district managers in five provinces, namely Mpumalanga, North West, Northern Cape, Limpopo and Eastern Cape, where the NHI sites were located. Informed consent was obtained from all the participants or respondents. Anonymity was ensured through the exclusion of personal details from the questionnaire and was further ensured via email correspondence, by sending it to the clinic administrator’s email address.

## Results

A total of 7 of the available 32 NHI pilot clinics (from two of the five provinces) responded to the invitation to be part of the research study, resulting in a 22% response rate. A total of 19 questionnaires were completed and returned via courier delivery. Reasons for non-consent to participation included the clinic manager being unavailable, no answer despite repeated calls or staff previously contacted no longer working at the site and electronic network challenges in specific areas.

Of the 19 respondents, all but 4 (79%) were professional nursing staff. Of these four respondents, one was a medical officer, one an ear nose and throat (ENT) specialist and two were operational managers. A total of 14 respondents (74%) indicated the number of years of experience in their profession. This provided a mean of 11 years, with a minimum of 1 year of experience, a maximum of 30 years of experience and a range of 29 years.

Of the 17 responses, the average number of babies seen per month ranged from 24 to 960 (see Table 1).

**TABLE 1:** Average number of babies seen at the primary healthcare clinics designated as National Health Insurance sites.

NHI site	NHI District	Type of clinic	Province	Number of respondents	Average number of babies seen per month
A	Pixley ka Seme	CHC	Northern Cape	4	30–50
B	Pixley ka Seme	PHC	Northern Cape	1	100
C	Pixley ka Seme	PHC	Northern Cape	2	24
D	Dr Kenneth Kaunda	PHC	North West	1	800
E	Dr Kenneth Kaunda	PHC	North West	5	960
F	Dr Kenneth Kaunda	PHC	North West	5	345
G	Dr Kenneth Kaunda	PHC	North West	1	345

CHC, community health centre; NHI, National Health Insurance; PHC, primary healthcare.

**FIGURE 1:**
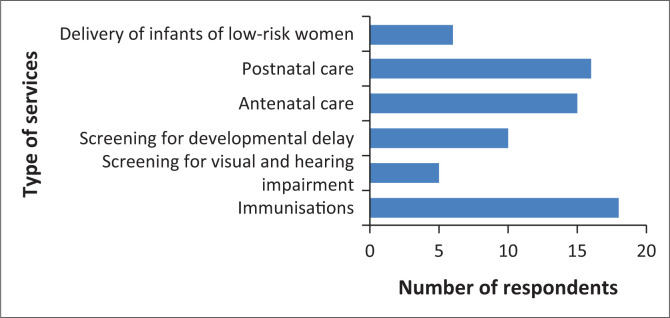
Types of services offered at primary healthcare facilities.

In terms of the types of services offered at the PHC facility, most respondents across all clinics, with the exception of Clinic D indicated immunisation services (*n* = 18; 95%), followed by post-natal care (*n* = 16; 84%) and antenatal care (*n* = 15; 79%). A total of 10 respondents (53%) indicated that screening for developmental delay is offered at the PHC facility at which they are employed, whereas services such as delivery of low-risk neonates and screening for visual and hearing impairment were only reported by 6 (32%) and 5 respondents (26%), respectively (see Figure 1). The six respondents were from clinics A, B and F, and the five respondents were from clinics A, B, F and G.

With regard to early detection of hearing loss in newborns and infants, 7 respondents (37%) from clinics A, B, F and G indicated that hearing screening services have been provided at their facility. In terms of the number of years that these services have been offered, two respondents indicated a period of more than 2 years, one indicated it being offered for more than 1 year, two for less than 6 months, one for less than a month and one respondent reported that these services were offered by the speech therapist who visited the site monthly but that there have been no services since 2018 as a result of no appointment of a therapist.

A total of 5 respondents (26%) from clinics A, F and G indicated these services to be in the form of universal newborn and infant hearing screening (UNIHS), whereas 3 respondents (16%) indicated screening based on parental or caregiver concern. One respondent from clinic A indicated a combination of UNIHS, risk-based NIHS and screening based on parental or caregiver concern.

In terms of equipment resources, only 2 respondents (11%) from clinics D and F indicated having an otoscope at their facility. The remaining respondents did not indicate having any equipment with one of these respondents mentioning conducting screening through communicating with the mother and baby and observing responses, whilst two other respondents mentioned using the IMCI protocols as a guide for screening.

With regard to staffing or human resources, 13 respondents (68%) provided information related to the number of nursing staff at their PHC facility. These numbers ranged from a minimum of 2 to a maximum of 15. Only 2 (11%) of these respondents (from clinics F and G) indicated having been trained to conduct NIHS. In terms of financial resources, only 2 respondents (11%), both from clinic F in the North West province indicated having a budget for NIHS but indicated that this budgetary amount is unknown.

When asked about the measures used to screen for hearing loss in low-risk or well babies, 2 respondents (11%) from clinics D and F indicated using an otoscope, whilst another two from clinics F and G mentioned the use of IMCI protocols for infants between 1 and 3 months of age. With regard to high-risk babies, more (*n* = 11; 58%) respondents indicated making use of the IMCI protocols to identify risk factors associated with hearing loss.

A total of 2 respondents (11%) from clinic A indicated that should infants be suspected to be at risk of hearing loss they would request a rescreening through referral to another PHC facility. A total of 3 respondents (16%) from clinics D and F indicated that they would refer for a diagnostic assessment, with one of these respondents (from clinic D) specifying this to be in the form of referral to an ENT. A further 7 (37%) respondents from clinics A, E, F and G indicated ensuring caregiver education in this regard. More specifically, in terms of follow-up and referral pathways for infants suspected to have hearing loss, 2 respondents (11%) from clinic F indicated booking follow-up appointments at the same PHC facility, 3 (16%) from clinics A and D indicated booking a follow-up at the nearest healthcare facility with a doctor, 5 (26%) from clinics D, E, F and G indicated referring to a healthcare facility with an ENT and 1 (5%) from clinic B to a nearest tertiary hospital. Options related to referral or follow-up at a healthcare facility with audiological services or to the next level of healthcare (i.e. district level) were not options considered by respondents.

## Discussion

This study aimed to establish whether NIHS could be integrated into the PHC re-engineering process as part of the NHI. This study’s findings are positioned in phases 1 and 2 of the NHI implementation plan. These phases have been planned to include piloting of different interventions in preparation for complete implementation of NHI, such as infrastructure and work streams, as well as the development and amendment to NHI and related legislation (Genesis et al., 2019). Despite these phases, healthcare professionals have expressed their concerns regarding the implementation of NHI. Findings from a recent survey study representative of 30 medical professions (including audiologists) indicated that they do not believe that the NHI will assist in improving service delivery and proposed that the healthcare system would need to be restored (Welthagen, 2019).

Survey respondents in this study primarily comprised professional nursing staff, followed by operational managers and medical doctors. Nurses are the largest category of healthcare workers in South Africa (WHO, 2017). Staffing models related to the PHC re-engineering in South Africa have suggested that all clinics should have a professional nurse to manage the facility (Daviaud & Subedar, 2012). Nurses form the backbone of PHC in South Africa and are made up of three cadres, namely professional nurses requiring 4 years of training, enrolled nurses requiring 2 years of training and nursing assistants requiring 1 year of training (WHO, 2017).

The average number of years of experience of respondents is lower than other studies involving professional nursing staff. Findings from a recent study by Muthathi, Levin and Rispel (2020) indicated the mean number of years of experience as 22 and 28 years in PHC clinics in Mpumalanga and Gauteng, respectively. Although direct comparison cannot be made to this study’s findings because of the inclusion of different provinces, findings suggest varying years of experience between and within provinces in South Africa. Despite the development of staffing models over the years, there are as yet no agreed national human resource norms for PHC in South Africa (WHO, 2017). This has implications for resourcing, service delivery and planning and ensuring appropriate staff:patient ratios.

As discussed previously, PHC facilities have been proposed as a platform for NIHS (HPCSA, 2007, 2018; Swanepoel et al., 2006), with the argument that this could facilitate coverage rate, particularly as PHC includes immunisations and post-natal care, as reported by respondents in this study. Despite this argument, only five respondents in this study indicated the inclusion of screening for hearing impairment of low-risk neonates or infants. When asked specifically about early detection of hearing impairment in newborns and infants, less than half of the respondents indicated recent implementation of hearing screening services, with varied responses within the same clinic. Although UNIHS was reported by some respondents in this study, implementation of these services is questionable; particularly as information pertaining to resources was suggestive of limited to no availability of appropriate hearing screening equipment. This has significant implications for the provision of valid and reliable NIHS and appropriate referral. The findings of this study are consistent with other studies conducted at PHC facilities in South Africa. A study conducted by Petrocchi-Bartal and Khoza-Shangase (2014b) with PHC nurses at immunisation clinics in the Northwest and Gauteng provinces revealed no formalised NIHS programmes, with cited key concerns related to limited training, budgetary, human resource and equipment constraints. Similar findings were reported by PHC nurses in KwaZulu-Natal. A total of 35 of the 75 nurses indicated the availability of otoscopes in comparison to 3 indicating the availability of a pure tone audiometer and tympanometer (Khan, Joseph, & Adhikari, 2018). This suggests the lack of, and an under-preparedness for, formalised NIHS at PHC facilities in South Africa, which may be attributed to the reported human resource and budgetary constraints.

The NHI Bill (Republic of South Africa, 2019) suggests the implementation of municipal ward-based teams, which include community healthcare workers. These community healthcare workers could be trained to facilitate NIHS service delivery at the primary healthcare level and ensure appropriate referral to higher levels of care. This shifting of tasks could also be a possible solution to addressing human resource constraints. Audiologists would need to be considered as part of the team in order to ensure efficient and effective management of such programmes. This is particularly important, as current procedures and protocols used at PHC facilities are inadequate for the early identification of hearing impairment (Khan et al., 2018).

Respondents in this study reported the use of observational responses to speech as a screening method and the use of the IMCI protocol as a screening guideline and to identify infants at risk for hearing impairment. Behavioural observation is no longer recommended in international guidelines as it is not a conditioned response and therefore does not yield reliable and valid information regarding hearing sensitivity (JCIH, 2019). The IMCI aims to decrease mortality, illness and disability and promote improved growth and development amongst children under 5 years of age (WHO, 2020). With regard to hearing, the IMCI protocol’s primary focus is on the detection of otitis media using questions, visual inspection and touch (DoH, 2019). Although otitis media is a risk factor for hearing impairment, there are other factors specified by the HPCSA (2018) EHDI guidelines that increase the risk of hearing impairment in newborns and infants. The use of the IMICI protocol has been reported in previous literature, along with the voice test (Khan et al., 2018; Petrocchi-Bartal & Khoza-Shangase, 2014b), all of which are subjective measures with notable limitations in terms of validity. With regard to follow-up and referral pathways for infants suspected to be at risk for hearing loss, a few respondents indicated referral to an ENT or nearest healthcare facility with a medical doctor, whilst seven mentioned caregiver education. Similar responses were noted in the study by Khan et al. (2018), whereby PHC nurses reported referrals to various health professionals, including ENT specialists and general practitioners and indicated provision of information about the importance of EHDI to parents or caregivers. However, contrary to this study’s findings, PHC nurses indicated referral to audiologists and paediatricians and specified referral to higher levels of care at the district hospital or clinic and paediatricians. Whilst referrals to healthcare professionals, particularly ENT specialists, are relevant, appropriate referral pathways are vital and need to be streamlined to ensure timely screening and diagnosis and to ensure continuity of care. Mukudu, Otwombe, Moloto, Fusheini and Igumbor (2021) compared self-referrals and headcounts at outpatient departments before and after the contracting of medical practitioners between pilot and non-pilot NHI districts. Findings indicated an increase in self-referrals and headcounts, resulting in a perceived improvement in quality care. However, structures need to be improved to ensure coordination between PHC and district level hospitals, by improving down-referral and the supervision of the PHC team by clinical specialists, which would further assist with the shortage of medical professionals.

Budgetary constraints and a lack of control over allocated funds have been reported by PHC managers in a recent study (Muthathi et al., 2020). Some facility managers reported a lack of transparency in terms of budget, whilst others reported to have no authority in decision making for the allocation of funds and provision of equipment. Similarly, two respondents in this study reported not knowing how much had been allocated to NIHS at their clinic. This has implications for resource allocation and the purchase of audiological screening equipment necessary for NIHS service delivery.

## Conclusions

Results of this study indicate limited to no implementation of NIHS at PHC NHI pilot sites. The availability of equipment and appropriate procedures and protocols is inconsistent despite government’s drive towards PHC re-engineering. Similarly, referral and follow-up practices for hearing screening are inconsistent, which impacts the continuity of care that is necessary for EHDI. There is a need for careful and systematic planning in terms of early hearing detection programmes at PHC level in order to ensure the provision of successful, streamlined services. This planning needs to commence with considerations of by whom NIHS will be performed, the training of these personnel by audiologists and the role of the audiologist within the teams outlined in the NHI Bill. As a result of the small sample size in this study and the poor response rate, findings cannot be generalised. Future research should be conducted on a larger sample size and should explore aspects related to governance, attitudes, infrastructure development and information systems.
